# Coronary microcirculation dysfunction evaluated by myocardial contrast echocardiography predicts poor prognosis in patients with ST-segment elevation myocardial infarction after percutaneous coronary intervention

**DOI:** 10.1186/s12872-022-02947-5

**Published:** 2022-12-28

**Authors:** Lan Wang, Yuliang Ma, Wenying Jin, Tiangang Zhu, Jing Wang, Chao Yu, Feng Zhang, Bailin Jiang

**Affiliations:** 1 Department of Cardiology, Peking University People’s Hospital, Beijing, China; 2 Beijing Key Laboratory of Early Prediction and Intervention of Acute Myocardial Infarction, Beijing, China; 3 Center for Cardiovascular Translational Research, Beijing, China; 4 Department of Anesthesiology, Peking University People’s Hospital, Beijing, China

**Keywords:** Acute ST-segment elevation myocardial infarction, Percutaneous coronary intervention, Myocardial contrast echocardiography, Coronary microcirculation, Major adverse cardiac events

## Abstract

**Background:**

The mortality rate of acute ST-segment elevation myocardial infarction (STEMI) remains substantial, despite advances in treatment strategies. Coronary microcirculation dysfunction (CMD) persists after percutaneous coronary intervention (PCI) in a substantial proportion of STEMI patients. The association between CMD assessed using myocardial contrast echocardiography (MCE) and prognosis requires further elucidation. This study aimed to evaluate the impact of CMD after successful PCI on the prognosis of patients with STEMI.

**Methods:**

We enrolled 167 patients with STEMI after PCI who underwent MCE during hospitalization between January 2018 and March 2022. Patients were classified into the CMD and non-CMD groups according to the results of MCE. The clinical data and MCE results of both groups were analyzed. Follow-up was conducted for major adverse cardiac events.

**Results:**

MCE detected CMD in 105 patients (62.9%). The CMD group contained fewer hypertensive patients (55.2% versus 74.2%, *P* = 0.015). Patients with CMD exhibited significantly higher levels of plasma troponin I (TnI) [73.2 (23.0–124.0) versus 28.9 (12.7–80.2) ng/mL, *P* = 0.004], higher levels of plasma B-type natriuretic peptide [255 (99–641) versus 193 (59–389) pg/mL, *P* = 0.004], poorer Killip classification (*P* = 0.038), and different culprit vessels (*P* < 0.001) compared to the non-CMD group. Patients with CMD exhibited lower left ventricular ejection fraction [50 (43–58) versus 61 (54–67) %, *P* < 0.001], poorer wall motion score index values (1.68 ± 0.4 versus 1.31 ± 0.26, *P* < 0.001) and poorer left ventricular global longitudinal strain [–11.2 (–8.7 to –14.1) versus –13.9 (–11.0 to –17.2) %, *P* < 0.001] compared to the non-CMD group. Patients underwent follow-up for 13 (7–20) months. After adjusting for hypertension, peak TnI level, culprit vessel, and Killip classification, CMD was an independent predictor of total major adverse cardiac events at 13 months’ follow-up [adjusted odds ratio (OR), 2.457; 95% confidence interval (CI), 1.042–5.790; *P* = 0.040], and patients with CMD had a higher risk of hospitalization for heart failure (adjusted OR, 5.184; 95% CI, 1.044–25.747; *P* = 0.044) and repeat myocardial infarction (adjusted OR, 2.896; 95% CI, 1.109–7.565; *P* = 0.030).

**Conclusions:**

MCE is a safe and effective method for detecting CMD in patients with STEMI. CMD detected by MCE after successful PCI in patients with STEMI is a common occurrence, which is associated with a significantly worse prognosis, especially hospitalization for heart failure and repeat myocardial infarction.

## Background

Acute ST-segment elevation myocardial infarction (STEMI), a frequent cause of hospital admission, is associated with significant short- and long-term mortality and morbidity. Although the acute and long-term mortality rates have fallen in parallel with the increase in the use of primary percutaneous coronary intervention (PCI), antithrombotic therapy, and secondary prevention, the mortality rate remains substantial. For example, the in-hospital mortality rate of patients with STEMI in the national registries of the member countries of the European Society of Cardiology (ESC) varies between 4 and 12%, while the reported 1-year mortality is approximately 10% [1]. Heart failure (HF) and repeat myocardial infarction (MI) are the most common sequelae encountered during long-term follow-up in patients with STEMI, which result in poor prognosis [1]. Therefore, it is important to investigate the factors that influence the long-term prognosis of patients with STEMI [2]. Coronary microcirculation hypoperfusion persists after PCI in a substantial proportion of patients with STEMI, despite restoration of the patency of epicardial coronary circulation. In addition to atherosclerotic disease of the epicardial coronary arteries, myocardial ischemia can also be caused by coronary microcirculation dysfunction (CMD). One study showed that no-reflow was among the determinants of left ventricular ejection fraction (LVEF) decline, and the latter could increase long-term mortality in young STEMI patients [3]. Thus, CMD can explain left ventricular (LV) dysfunction and poor prognosis [4].

Numerous studies have used cardiovascular magnetic resonance (CMR) as a non-invasive tool for detecting CMD, although CMR has some limitations for patients with STEMI. Myocardial contrast echocardiography (MCE) is an established technique for the assessment of myocardial perfusion. The contrast agents in this technique are microbubbles approximately the size of red blood cells (< 7 μm in diameter). The myocardial signal intensity emanating from the contrast agent reflects the concentration of microbubbles within the myocardium. When the myocardium is fully saturated during continuous infusion of microbubbles, the signal intensity reflects the relative capillary blood volume. Any decrease in myocardial blood flow prolongs the replenishment time in proportion to the reduction in myocardial blood flow. Therefore, the European Association of Cardiovascular Imaging (EACI) recommends the use of contrast agents for the assessment of myocardial perfusion [5]. MCE has found widespread application owing to its non-invasiveness, lower cost, ease of implementation, and greater patient acceptance. The association between CMD assessed by MCE and prognosis has recently garnered interest [6, 7]. Therefore, we designed this study with the aim to evaluate the impact of CMD after successful PCI on the prognosis of patients with STEMI using MCE.

## Methods

### Participants

All consecutive patients with STEMI who underwent MCE after PCI during hospitalization at Peking University People’s Hospital between January 2018 and March 2022 were enrolled in this study. The patients were diagnosed with STEMI after an expert physician review based on the fourth universal definition of myocardial infarction published in 2018 [8]. Patients who did not provide informed consent, those who did not undergo MCE during hospitalization, or were allergic to sulfur hexafluoride (Sono-Vue) were excluded from the study during the initial stage. Patients were subsequently categorized into the CMD and non-CMD groups based on the results of MCE. The MCE data of the two groups of patients were analyzed and compared. Demographic parameters including age, sex, body mass index, and clinical characteristics including history of smoking, hypertension, diabetes mellitus, laboratory data, coronary angiography data, and revascularization data were extracted. Venous blood samples were collected for analysis. Troponin I (TnI) and B-type natriuretic peptide (BNP) were analyzed using chemiluminescence immunoassays. The study was approved by the Ethics Committee of Peking University People’s Hospital (ethics committee approval number: 2022PHB116-001) and informed consent was obtained from all patients.

### Echocardiography and MCE

**Fig. 1:**
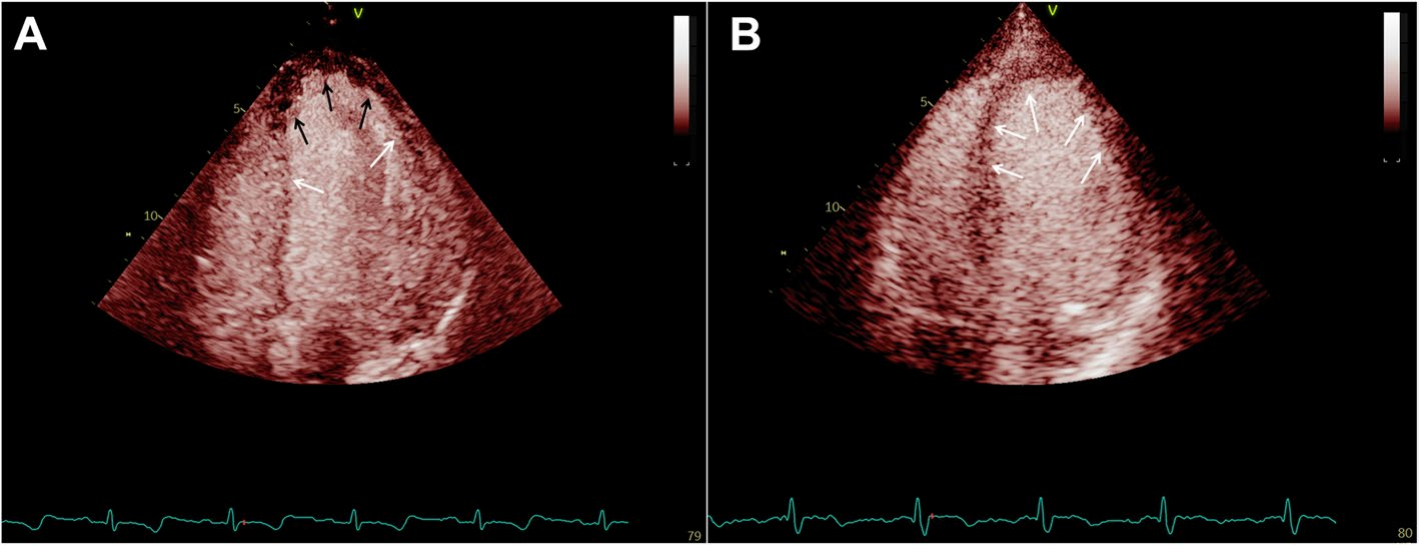
Examples of abnormal microvascular perfusion and normal microvascular perfusion. Demonstration of abnormal (**A**) and normal (**B**) myocardial contrast replenishment on real-time myocardial contrast echocardiography Black arrows: myocardial perfusion defect; white arrows: normal myocardial perfusion

All patients underwent echocardiography and MCE within 7 days of PCI. Echocardiography was performed using a Vivid E95 Console Ultrasound Machine (GE Healthcare, USA). We used an M5Sc transducer with a 2.5–3.5 MHz imaging frequency for each study. Examinations were performed for at least three consecutive cardiac cycles. According to the 2015 American Society of Echocardiography (ASE) and European Association of Cardiovascular Imaging (EACI) echocardiography guidelines [9], the internal dimensions were obtained using 2-dimensional (2D) echocardiography and LVEF was measured using Simpson’s method. The ASE 17-segment LV model was used to analyze regional wall motion (WM); the segments were scored as normal (score = 1), hypokinetic (score = 2), akinetic (score = 3), dyskinetic (score = 4), or ventricular aneurysm (score = 4), and the average of the 17 segments was calculated to derive the WM score index (WMSI). LV global longitudinal strain (GLS) was measured using three standard apical views (apical long axis, four-chamber, and two-chamber). Speckles were visualized via frame-by-frame tracking throughout the LV wall during the cardiac cycle, and basal, mid, and apical regions of interest were created. Segments that could not be tracked automatically were adjusted manually by the operator, and the GLS was calculated. MCEs were performed in three-standard apical (four-, two-, and three-chamber) views using sulfur hexafluoride (Sono-Vue; Bracco International B.V., Italy). Sulfur hexafluoride (Sono-Vue) was dissolved in saline (5 mL), followed by slow intravenous injection (1 mL/min) and saline flushing (5 mL). Chamber opacification and WM were observed in the LV opacification mode, and myocardial perfusion was observed in MCE mode. Real-time imaging with brief high-mechanical-index impulses was performed for 15 continuous cardiac cycles to analyze replenishment. We also implemented a 17-segment model as described by the ASE/EACI [9] and points were allocated if the segment was completely replenished with the contrast in the myocardium within 4 s (1 point), 4–10 s (2 points), and > 10 s (3 points). The myocardial perfusion index (MPI) was derived from the average of the 17 segments (Fig. 1). An MPI score of 1 was designated as non-CMD, while an MPI score > 1 was designated as CMD.

### Follow-up and definition of major adverse cardiac events

The participants’ electronic medical records were reviewed to identify major adverse cardiac events (MACEs). Death, hospitalization for HF, or repeat MI were denoted as MACEs [10]. The diagnosis of HF was based on the 2021 universal definition and classification of HF [11]. The diagnosis of MI was based on the 2018 fourth universal definition of myocardial infarction [8].

### Statistical analysis

According to a previous study [10], the incidence of hospital admission for HF in patients with normal perfusion and CMD was 2% and 18%, respectively. We estimated that 99 patients would be required in the CMD group and 49 patients in non-CMD group to attain a statistical power of 80% to show a significant difference between the groups with a two-sided α level of 5%, and attrition rate of 20%.

All statistical analyses were performed using SPSS software (Version 21.0, IBM, Armonk, New York, USA). Continuous variables were presented as the mean ± standard deviation (SD) for normally distributed variables and as the median (interquartile range) for non-normally distributed variables. Categorical variables were described as numbers (percentages). Associations among variables were evaluated using the t-test, Mann–Whitney U test, and χ^2^ tests. The Spearman correlation test was used to analyze the correlation between the MPI and GLS. Cox survival analysis was performed to detect the effect of CMD on MACEs. Statistical significance was set at *P* < 0.05.

## Results

### Baseline characteristics

A total of 167 patients who developed STEMI after PCI were enrolled in this study. MCE detected CMD in 105 patients (62.9%). There were fewer patients with hypertension in the CMD group (55.2% versus 74.2%, *P* = 0.015). Patients with CMD exhibited significantly higher levels of plasma TnI [73.2 (23.0–124.0) versus 28.9 (12.7–80.2) ng/mL, *P* = 0.004], higher levels of plasma BNP [255 (99–641) versus 193 (59–389) pg/mL, *P* = 0.004], poorer Killip class (*P* = 0.038), and different culprit vessels (*P* < 0.001). The proportion of successful thrombolysis in the CMD and non-CMD groups was 21.0% and 21.0%, respectively (*P* = 0.887). The time required for symptom-to-flow restoration (PCI or thrombolysis) in CMD and non-CMD patients was 6.7 h and 5.7 h, respectively (*P* = 0.814). There were no differences in the other clinical data (Table 1). Blood flow in all culprit vessels was restored to Thrombolysis In Myocardial Infarction (TIMI) grade 3 after PCI.

**Table 1:** Baseline characteristics of participants

Variable	CMD group (*n* = 105)	Non-CMD group (*n* = 62)	*P*
Male (%)	85 (81.0)	48 (77.4)	0.584
Age, years	59 (47–69)	58 (54–68)	0.280
Smoker (%)	62 (59.0)	38 (61.3)	0.775
BMI, kg/ m2	25.3 ± 3.8	25.8 ± 3.3	0.342
Hypertension (%)	58 (55.2)	46 (74.2)	0.015
Diabetes (%)	34 (32.4)	23 (37.1)	0.535
Peak troponin I, ng/ mL	73.2 (23.0–124.0)	28.9 (12.7–80.2)	0.004
B-type natriuretic peptide, pg/ mL	255 (99–641)	193 (59–389)	0.032
C-reactive protein, mg/ L	4.3 (0.7–32.8)	2.6 (0.5–11.5)	0.145
LDL-C, mmol/ L	2.93 (2.26–3.62)	2.85 (2.42–3.46)	0.866
Fasting plasma glucose, mmol/ L	6.06 (4.97–7.43)	5.91 (5.18–6.85)	0.999
eGFR, ml/min/1.73m^2^	90.0 (73.5–102.1)	82.7 (66.4–98.6)	0.081
Killip classification			0.038
Grade 1 (%)	82 (78.1)	58 (93.5)	
Grade 2 (%)	15 (14.3)	3 (4.8)	
Grade 3 (%)	2 (1.9)	0 (0.0)	
Grade 4 (%)	6 (5.7)	1 (1.6)	
Time of symptom-to-flow restored (PCI or thrombolysis), h	6.7 (3.5–22.7)	5.7 (3.7–32.2)	0.814
Thrombolysis (%)	23 (21.9)	13 (21.0)	0.887
Culprit vessel			< 0.001
LAD (%)	69 (65.7)	22 (35.5)	
LCX (%)	12 (11.4)	8 (12.9)	
RCA (%)	24 (22.9)	32 (51.6)	
Multivessel disease	79 (75.2)	55 (88.7)	0.056

Data are expressed as mean ± SD, number (percentage), or median (interquartile range). *BMI* Body mass index, *CMD* Coronary microcirculation dysfunction, *eGFR* Estimated glomerular filtration rate, *LAD* left anterior descending artery, *LCX* left circumflex coronary artery, *LDL-C* low-density lipoprotein cholesterol, *PCI* percutaneous coronary intervention, *RCA* right coronary artery

### Echocardiography and MCE

Patients with CMD had lower LVEF [50 (43–58) versus 61 (54–67), *P* < 0.001], poorer WMSI values (1.68 ± 0.4 versus 1.31 ± 0.26, *P* < 0.001) and poorer left ventricular GLS [–11.2 (–8.7 to –14.1) versus –13.9 (–11.0 to –17.2) %, *P* < 0.001] (Table 2). The median MPI in patients with CMD was 1.24 (1.12–1.52). MPI was positively correlated with GLS in patients with CMD (r = 0.585, *P* < 0.001).

**Table 2:** Echocardiogram and MCE parameters between the groups

	CMD group (*n* = 105)	Non-CMD group (*n* = 62)	P
LVEDd, cm	5.0 ± 0.6	4.9 ± 0.5	0.460
LVEF, %	50 (43–58)	61 (54–67)	< 0.001
RWMA (%)	101 (96.2)	52 (83.9)	0.006
WMSI	1.68 ± 0.4	1.31 ± 0.26	< 0.001
GLS, %	-11.2 (-8.7 to -14.1)	-13.9 (-11.0 to -17.2)	< 0.001

Data are expressed as mean ± SD or number (percentage), *CMD* coronary microcirculation dysfunction, *GLS* global longitudinal strain, *LVEDd* left ventricular end-diastolic dimension, *LVEF* left ventricular ejection fraction, *MCE* myocardial contrast echocardiography, *RWMA* regional wall motion abnormality, *WMSI* wall motion score index

### Clinical follow-up

**Fig. 2:**
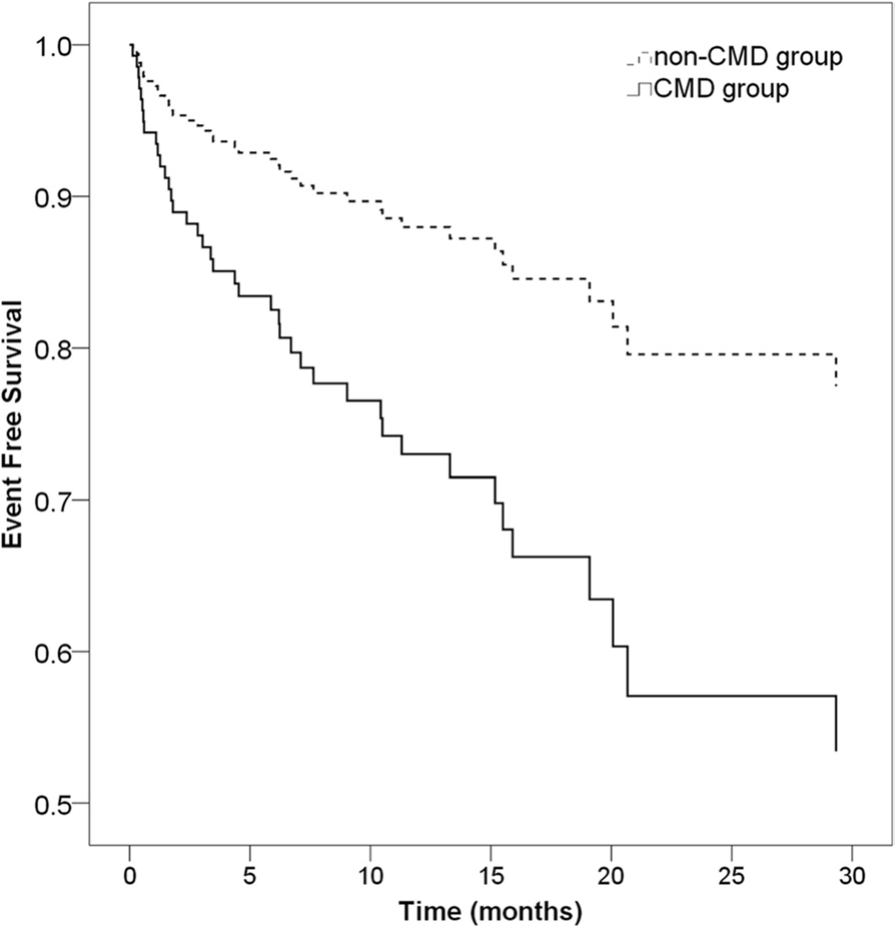
Event-free survival for all MACEs. The frequency of MACEs was higher in patients with CMD during 13 months of follow-up (OR: 2.457, 95%CI: 1.042–5.790, *P* = 0.040). MACE: major adverse cardiac event, CMD: coronary microcirculation dysfunction

Patients underwent follow-up for 13 (7–20) months. CMD was found to be an independent predictor of all MACEs at 13 months’ follow-up [adjusted odds ratio (OR), 2.457; 95% confidence interval (CI), 1.042–5.790; *P* = 0.040; Fig. 2], after adjusting for hypertension, peak TnI level, culprit vessel, and Killip classification,. Patients with CMD were at a higher risk of hospitalization for HF (adjusted OR, 5.184; 95% CI, 1.044–25.747; *P* = 0.044) and repeat MI (adjusted OR, 2.896; 95% CI, 1.109–7.565; *P* = 0.030; Table 3).

**Table 3:** MACEs of the two groups of patients

MACEs	CMD group (*n* = 105)	Non-CMD group (*n* = 62)	OR value (95%CI)	P
Death	2 (1.9%)	0 (0)	261,866.8(/)	0.973
Hospitalization for HF	14 (13.3%)	2 (3.2%)	5.184(1.044–25.747)	0.044
Repeat MI	21 (20.0%)	7 (11.3%)	2.896 (1.109–7.565)	0.030

Data are expressed as number (percentage), *CMD* coronary microcirculation dysfunction, *HF* heart failure, *MACE* major adverse cardiac events, *MI* myocardial infarction

## Discussion

The vascular compartment in the myocardium comprises the great arteries, arterioles, capillary network and smaller intra-myocardial veins. The coronary microcirculation is characteristically composed of vessels smaller than 200 µm in diameter. The microcirculation not only serves as a passive channel through which blood is transported into the myocardium, but rather an active site of blood flow control with complex metabolic and myogenic regulatory mechanisms. Currently, early mechanical reperfusion of the epicardial coronary artery by PCI is the recommended therapy for STEMI. Successful restoration of epicardial coronary blood flow is achieved in over 95% of PCI procedures, but complete restoration of perfusion of the distal coronary microvasculature is not achieved, even though angiographic culprit blood flow is reperfused to TIMI 3 grade in approximately half of the patients, leading to CMD [12]. Several mechanisms are involved in the development of CMD after STEMI, including ischemia-related injury, reperfusion-related injury, distal embolization, intramyocardial inflammation, and individual susceptibility of the microcirculation to injury [6, 13]. Therefore, evaluation of CMD is necessary since it may affect the prognosis of STEMI.

Various diagnostic modalities can be used for the assessment of CMD, including both non-invasive and invasive techniques. Each methods has its own strengths and weaknesses [7]. Studies have shown that the index of microcirculatory resistance (IMR) and hyperemic microvascular resistance (HMR) are useful for the early identification of severe CMD in patients with STEMI after PCI, which are associated with a higher risk of long-term MACEs in patients with STEMI [14]. However, both IMR and HMR are invasive. The systemic inflammation marker, i.e., the ratio of serum C-reactive protein to albumin, has been proven to predict imperfect reperfusion that can worsen the prognosis of STEMI [15]. Several studies have frequently used CMR as a non-invasive tool for detecting CMD [16]. Nevertheless, CMR also has some limitations, such as prolonged offline post-processing, concerns over the safety of gadolinium-based contrast agents, and association with nephrogenic systemic fibrosis in patients with chronic kidney disease. In our study, MCE was chosen as a non-invasive tool to detect CMD in patients with STEMI. Moreover, MCE is a more convenient, economical, and safer method that can accurately identify patients who develop CMD after STEMI.

The principle underlying MCE entails the administration of microbubble contrast agents via the peripheral veins to improve the echocardiographic signal. Ultrasound-induced microbubble destruction can be used for the measurement of myocardial blood flow during continuous infusion of contrast agents. The relative agent concentration in different myocardial beds represents the capillary density or the sum of its cross-sectional area. The microbubble concentration in the microcirculation reflects the blood volume in the microvasculature. Thus, MCE facilitates effective assessment of the coronary microcirculation. Studies have shown that the incidence of CMD after STEMI is approximately 54.9–89% (10.12.13). Our study indicated that 62.9% of patients developed CMD, despite the success of PCI. We observed a similar incidence of CMD based on MCE examination. Thus, we can infer that MCE is a reliable tool to detect CMD, and that CMD is a common phenomenon that warrants attention.

In this study, patients with CMD exhibited significantly higher levels of TnI and BNP, poorer Killip class, and different culprit vessels. These results suggest that CMD may be associated with the degree of myocardial necrosis and cardiac function during the acute phase of STEMI. One study showed that the Killip classification can predict the risk of CMD in patients with STEMI undergoing PCI [17]. Meanwhile, MCE revealed that the LVEF and GLS were lower and WM was worse in patients with CMD. Our previous study showed that CMD was independently associated with the global LV myocardial work index assessed by echocardiography (adjusted OR 0.997, 95% CI 0.994–1.000, *P* = 0.029) [18]. Another of our previous studies found that CMD could lead to exacerbation in the LVEF and WM in patients with STEMI, after adjusting for culprit vessels [19]. Moreover, CMD after acute MI increases the risk of acute HF during hospitalization [20]. These results suggest that CMD after STEMI can result in a poor prognosis during hospitalization.

We found that CMD was an independent predictor of total MACEs at 13 months of follow-up, after adjusting for hypertension, peak TnI level, culprit vessel, and Killip classification. The risk of re-hospitalization-adjusted HF increased more than fourfold (OR, 5.184; *P* = 0.044), while that of repeat MI increased nearly two-fold (adjusted OR, 2.896; *P* = 0.030). Animal studies have demonstrated a direct association between persistent microvascular obstruction and adverse ventricular remodeling [21]. The presence of microvascular obstruction is a powerful predictor of LV remodeling and HF events [6]. Post-ischemic CMD is predictive of a greater than fourfold increase in the long-term risk of adverse outcomes, which is mainly driven by the occurrence of HF [22]. In this study, MCE confirmed that the risk of HF could still increase in the presence of CMD, despite complete timely revascularization (the median revascularization time was 6.7 h in the CMD group). HF is a common complication of STEMI and is predictive of a poor prognosis [1]. Although national door-to-balloon times have improved significantly over the last few years for patients undergoing primary PCI, several STEMI patients still develop HF in the long term. Identification and intervention of CMD may provide new concepts for the prevention of HF after STEMI. This study also found that patients with CMD were at a higher risk of repeat MI (adjusted OR, 2.896; *P* = 0.030). It is generally assumed that epicardial events precede and cause CMD. However, a novel concept of the pathogenic mechanism was posited, which states that transient or permanent microvascular dysfunction limits coronary blood flow, leads to alterations in shear stress (affecting endothelial function), and enhances thrombus formation at the epicardial level [7]. This may explain the high risk of long-term repeat MI to some extent. However, the existence of other mechanisms requires further investigation. Subgroup analysis of the FOURIER study showed that patients with a history of repeat MI were at a higher risk of MACEs and a poor prognosis [23]. These findings underscore the necessity of identifying CMD in patients with STEMI.

To date, no randomized trials have compared therapies for the reduction of adverse cardiac events in patients with CMD. Lifestyle changes and risk factor management should be considered essential in the management of CMD. Conventional pharmacotherapeutic strategies, such as beta-blockers, angiotensin-converting enzyme inhibitors (ACEIs) or angiotensin receptor blockers (ARBs), statins, antiplatelet therapy and nitrates are recommended [7]. Research into novel treatment for CMD is an unmet clinical need. One novel strategy entails inhibiting Rho-kinase in order to ameliorate CMD [24]. Therapeutic modalities targeting perivascular adipose tissue to stimulate the production of vasoactive and vasorelaxant factors such as adiponectin or hydrogen sulfide could be beneficial [25, 26]. All patients enrolled in our study had received standard conventional therapy for coronary heart disease. Further studies focusing on the treatment and management of patients with CMD remain a clinical requirement.

A previous study found that CMD was associated with long-term mortality in patients with STEMI [10]. However, we did not find any difference in the risk of mortality between the CMD and non-CMD groups (*P* = 0.973). The sample size of our study was merely 167 patients, and the follow-up period was relatively short; only two patients died in the CMD group, and none died in the non-CMD group; thus, the mortality-related data did not converge in the Cox regression analysis. Moreover, we only analyzed the culprit vessels and multivessel disease, whereas the effect of other coronary anatomical data on perfusion was not compared. The sample size and short follow-up period were limitations of our study and may be responsible for the lack of significant differences in the mortality between the two groups. Future studies with larger samples and longer follow-up durations should be performed and more coronary anatomical data should be analyzed. Moreover, the change in coronary microcirculation perfusion is a dynamic process, but we only obtained MCE data at one period of time (within 7 days post-PCI). Imaging data at follow-up should be added to gain a more comprehensive understanding of the impact of CMD on STEMI.

We used MCE to detect CMD, which is a safer and more convenient method for patients with STEMI. Our findings revealed that CMD occurred in a significant proportion of patients with STEMI, which led to a poor prognosis. This study provides evidence of the feasibility of predicting MACEs using MCE in patients with STEMI and proved the importance of early identification of CMD in patients with STEMI.

## Conclusions

CMD in patients with STEMI is common and associated with a significantly worse prognosis despite successful PCI, resulting in hospitalization for HF and repeat MI.

## Data Availability

The datasets used and/or analyzed during the current study are available from the corresponding author on reasonable request.

## Abbreviations

2D: 2-Dimensional
ASE: American Society of Echocardiography
BNP: B-type natriuretic peptide
CMD: Coronary microcirculation dysfunction
CMR: Cardiovascular magnetic resonance
EACI: European Association of Cardiovascular Imaging
eGFR: Estimated glomerular filtration rate
GLS: Global longitudinal strain
HF: Heart failure
HMR: Hyperemic microvascular resistance
IMR: Index of microcirculatory resistance
LDL-C: Low-density lipoprotein cholesterol;
LV: Left ventricular
LVEDd: Left ventricular end-diastolic dimension
LVEF: Left ventricular ejection fraction
MACE: Major adverse cardiac event
MCE: Myocardial contrast echocardiography
MI: Myocardial infarction
MPI: Myocardial perfusion index
OR: Odds ratio
PCI: Percutaneous coronary intervention
LAD: Left anterior descending artery
LCX: Left circumflex coronary artery
RCA: Right coronary artery
RWMA: Regional wall motion abnormality
STEMI: ST-segment elevation myocardial infarction
TnI, troponin I: TIMI: Thrombolysis In Myocardial Infarction
WM: Wall motion
WMSI: Wall motion score index
